# Cost estimation and planning for HIV/AIDS oral healthcare services: a Malaysian experiment

**DOI:** 10.1186/1471-2334-12-S1-P93

**Published:** 2012-05-04

**Authors:** Chen-Chen Yong, Jacob John, Evangelos Koutronas, Thomas George Kallarakkal

**Affiliations:** 1 Faculty of Economics and Administration, University of Malaya, Kuala Lumpur, Malaysia; 2 Faculty of Dentistry, University of Malaya, Kuala Lumpur, Malaysia

## Background

Reuters reported that 8400 HIV cases were detected in the voluntary screening of drug addicts in Harm Reduction Programmes, and screening in prison and Narcotics Rehabilitation Centers in 2011. However, dental treatment for HIV inmates is highly constrained by high uncertainty resulting from the changes of epidemic profile after receiving medical treatment, relative inadequacy of dental treatment and rules and regulations. Thus institutional HIV dental care cost could pose challenges to government healthcare expenditure.

## Methods

The marginal cost for dental treatment is estimated based on the case scenario of prison X in Malaysia. Based on literature review and economic reasoning, an integrated cost planning model is formed to address the current needs in cost planning.

## Results

The marginal cost for prison dental treatment is estimated to increase by at least 3 folds for prevalence cases and by at least 7 folds for surveillance case in short run. The cost pyramid is formed with the base of cost-consequences, cost efficiency and cost effectiveness planning followed by cost benefit and financial risk planning at the second layer, and cost utility planning at the peak layer. All cost planning components are integrated vertically and horizontally.

## Conclusion

HIV dental care costs for inmates will be at the expense of government revenue if the increased marginal cost is above the average cost. Therefore, cost estimation coupled with the integrated cost planning model is essential for evaluation, monitoring and budget allocation enhancement.

